# Design of Biocompatible Chitosan/Polyaniline/Laponite Hydrogel with Photothermal Conversion Capability

**DOI:** 10.3390/biom12081089

**Published:** 2022-08-07

**Authors:** Liying Zhang, Gao He, Yang Yu, Yu Zhang, Xiang Li, Shige Wang

**Affiliations:** 1 School of Materials and Chemistry, University of Shanghai for Science and Technology, No. 516 Jungong Road, Shanghai 200093, China; 2 The First Clinial Medical College, Nanjing Medical University, No. 101 Longmian Road, Nanjing 211166, China

**Keywords:** hydrogel, chitosan, polyaniline, photothermal, biocompatibility

## Abstract

In recent years, multifunctional hydrogels have received a great deal of attention because they are biocompatible and can mimic the extracellular matrix. Herein, we prepared hydrogels of biocompatible cross-linked networks with photothermal properties. In this study, a chitosan/polyaniline/laponite (COL) hydrogel with photothermal conversion capability was designed. Polyaniline was firstly grafted onto chitosan and its solution was mixed with oxidized dextran, which was then cross-linked into a hydrogel via a Schiff base reaction. Furthermore, an aluminosilicate clay material, laponite (LAP), was incorporated into the hydrogel. The swelling ratio of the COL hydrogel in various solutions was greater than 580%, and it showed good degradation ability (the mass–loss ratio was over 45% after 28 days). This composite hydrogel was demonstrated to have good photothermal conversion properties and biocompatibility at both the cell (cell viability was over 97%) and animal levels. The COL hydrogel showed a photothermal conversion efficiency of 23.7% under the irradiation of a near-infrared laser. Coupled with the osteogenic differentiation-inducing potential of LAP, the COL hydrogel has the potential to kill tumors via hyperthermia or serve as scaffolds for bone tissue regeneration.

## 1. Introduction

Photothermal therapy (PTT) is an effective, non-invasive, non-systemic tumor therapy method, especially for the local treatment of superficial tumors [1,2]. In the process of PTT, the photothermal agent is first concentrated on the lesion, and then near-infrared (NIR) light is used to irradiate the lesion. Elevated temperatures kill tumor cells while avoiding side effects on normal cells [3]. An ideal photosensitizer should have strong absorbance in the NIR region and be able to effectively convert the absorbed NIR light energy into heat energy [4]. In addition, it should have good biocompatibility. Currently, the main conjugated polymers (CPs) used for tumor PTT include polyaniline (PAI), polypyrrole, and polydopamine [5]. PAI has a large π–π conjugated skeleton structure, which can absorb infrared light for photothermal conversion [6]. Biocompatible hydrogels are composed of hydrophilic polymers, which can absorb large amounts of water or biological fluids and maintain a unique three-dimensional network structure. Hydrogels have been frequently studied as scaffolds, the core of bone tissue engineering (BTE) [7,8].

Chitosan (CS) is the deacetylated product of chitin that exists in the shells of insects, mollusks, and crustaceans, as well as in bacterial and fungal cell walls [9]. CS is a polysaccharide containing cleavable glycosidic bonds [10], which helps CS to be biocompatible and degradable, as well as to mimic the constituents of the extracellular matrix and provide suitable conditions for cells to survive in scaffold materials [11,12]. In addition, it has been proven that CS has osteoconductive properties [13,14]. By its hydroxyl and free amine groups, CS can be modified differently according to requirements [15]. CS-based hydrogels have the advantages of simple preparation, low cost, high conductivity, and good biocompatibility [16]. At present, CS-based hydrogels have been dedicated to improving different applications such as BTE, in situ tumor therapy, and wound dressings [14,17,18,19].

Based on these properties, we herein designed polyaniline-grafted chitosan aimed to combine the electrical conductivity and photothermal properties of PAI with the biocompatibility of CS. To introduce the necessary network, dextran (Dex) was oxidized to oxidized dextran (OD) and cross-linked with CS. In this study, aniline was firstly grafted onto CS and then gradually oxidized into PAI; therefore, compared to the direct physical blending of PAI, the blending uniformity of PAI could be improved [20,21]. Furthermore, to broaden the application potential of the hydrogel, an aluminosilicate clay that has been commonly used as an osteogenic additive, laponite (LAP) [22], was physically doped in the hydrogel. Owing to the conjugations between the aniline–CS and OD–CS, the density of amino groups in the hydrogel was reduced. Therefore, the hydrogel showed excellent biocompatibility. For example, macromolecules with multiple amino termini can be modified with poly-ethylene glycol to reduce their amino group densities and improve their biological safety [23]. Moreover, the introduction of PAI brought photothermal conversion capability to the hydrogel. Therefore, the hydrogel holds a promising application future as a tumor inhibitor with photothermal conversion ability.

## 2. Experimental Section

### 2.1. Materials

All chemicals were used without further purification. LAP RDS (Na_0.3_Mg_2.7_Li_0.3_Si_4_O_10_(OH)_2_) was purchased from the Zhejiang Institute of Geology and Mineral Resources, China. Dex, sodium periodate (NaIO_4_), CS, hydrochloric acid (HCl), aniline, ammonium persulfate, acetic acid, sodium hydroxide, dimethyl sulfoxide, and sodium bromide were bought from Shanghai Aladdin Bio-Chem Technology Co., Ltd., China. Ethanol and ethylene glycol were bought from Shanghai Yuanye Bio-Technology Co., Ltd., China. A phosphate buffer solution (PBS), citrate buffer solution (CBS), Dulbecco’s modified eagle medium (DMEM), and Live/Dead kit were purchased from Sigma-Aldrich (Shanghai) Trading Co., Ltd., China. The cell counting kit-8 (CCK-8) was purchased from Guangzhou Weijia Technology Co., Ltd., China. Mouse fibroblasts (L929) were obtained from the Institute of Biochemistry and Cell Biology, Chinese Academy of Sciences (Shanghai, China). Kunming (KM) mice (female, 4–6 weeks, 20–25 g) were ordered from the Shanghai Slac Laboratory Animal Center (Shanghai, China).

### 2.2. Preparation of Polyaniline-Grafted Chitosan

Typically, 1.2 g of CS was added to 120 mL of HCl (0.1 mol/L) and heated to 35 °C. Then, after obtaining the chitosan solution, 40 μL of aniline was added to the dissolved solution, and it was stirred in the dark for 2 h at room temperature. After the abovementioned operation, 90 mg of ammonium persulfate was added to the solution, and the reaction was initiated and continued at 25 °C for 24 h in the dark. After the reaction, the pH of the solution was adjusted to neutrality with an aqueous solution of sodium hydroxide (1 mol/L), and then excess ethanol was added to precipitate the synthesized product [24]. The final system was centrifuged at 8000 rpm/min for 5 min, and the solid phase was washed three times with dimethyl sulfoxide. The washed product was redispersed in deionized water (DI water) and dialyzed (MWCO: 14,000 Da, Viskase, IL, USA) for 3 days to remove organic solvents and impurities. The obtained pure product was freeze-dried to obtain polyaniline-grafted chitosan (CSP) and stored in a refrigerator at 4 °C for later use.

### 2.3. Preparation of Oxidized Dextran

OD was synthesized by a redox reaction between Dex and sodium periodate [8]. We added 10.0 g of Dex to 100 mL of DI water. After that, 13.0 g of sodium periodate was added to the solution, and the reaction mixture was stirred at 50 °C for 3 h. After the reaction, 4 mL of ethylene glycol was added to the solution to stop the dextran oxidation or to consume the unreacted periodate, and then the product was dialyzed against DI water (MWCO: 3500 Da, Viskase, IL, USA) and lyophilized to obtain dried solid OD that was stored in a 4 °C refrigerator for further use. The oxidation degree of OD was determined by the hydroxylamine hydrochloride titration method. In this method, the aldehyde groups of OD react with the amino groups of hydroxylamine hydrochloride to form oxime and simultaneously release HCl. The concentration of the aldehyde group can be calculated by titrating the amount of HCl, and the oxidation degree of OD can be determined accordingly. Briefly, OD was added to a 0.25 mol/L hydroxylamine hydrochloride aqueous solution (containing 5% methyl orange). This solution was titrated with 0.1 mol/L sodium hydroxide until the color turned yellow (suggesting the titration endpoint) [25,26]. The formula for calculating the OD oxidation degree was:

(1) $$\mathrm{Oxidation}\;\mathrm{degree}\;(\%)=\frac{(V_{NaOH}-V_{blank})\times M_{NaOH}\times M_{W_{Dex}}}{1000\times W_{OD}}\times 100$$

where $V_{NaOH}$ is the volume of NaOH consumed for the titration reaction, $V_{blank}$ is the volume of NaOH consumed in the control group, $M_{NaOH}$ is the molarity of NaOH, $M_{WDex}$ is the molecular weight of the DEX monomer, and $W_{OD}$ is the weight of OD.

### 2.4. Synthesis and Characterizations of COL Hydrogel

We mixed 0.05 g of OD and 0.06 g of LAP in 1 mL of water and stirred them until the final system became homogeneous. Meanwhile, 0.02 g of CSP was dissolved in 1 mL of a 0.5% acetic acid solution [27]. These two aqueous systems were mixed and stirred at room temperature for 10 s to obtain the COL hydrogel. The morphology of the prepared hydrogels and their surface composition were characterized with scanning electron microscopy (SEM, Zeiss Sigma 300, Oberkochen, Germany) and X-ray energy-dispersive spectrometry (EDS, Zeiss Sigma 300, Oberkochen, Germany). The hydrogels were lyophilized for 12 h, sliced into thin slices with a razor blade, and pasted on a conductive adhesive. To avoid degrading under the electron beam exposure, an ultrathin layer of gold was deposited on the material surface. The functional groups in the hydrogel were identified with Fourier transform infrared (FTIR, Nicolet Nexus 470) spectroscopy. The structural characterization of the hydrogels was performed with proton nuclear magnetic resonance (^1^H-NMR, Bruker 400 M, Switzerland) spectroscopy (400 MHz) by using deuterated water as the solvent for sample preparation.

### 2.5. Swelling Analysis of COL Hydrogel

For the swelling study, a piece of lyophilized hydrogel was weighed (W_0_) and immersed in PBS (0.01 mol/L, pH = 7.4), DMEM, or CBS (0.01 mol/L, pH = 5.4). After 24 h of immersion, when equilibrium swelling was reached, the resulting hydrogel was taken out (the water excess was gently removed by using a filter paper) and then weighed [28]. The swelling ratio of the hydrogel was calculated using the following formula:

(2) $$\mathrm{Swelling}\;\mathrm{ratio}\;(\%)=\frac{W_{1}-W_{0}}{W_{0}}\times 100$$

where $W_{1}$ is the maximum weight of hydrogel at equilibrium swelling and $W_{0}$ is the weight of the freeze-dried hydrogel before its swelling.

### 2.6. Degradation Analysis of COL Hydrogel

An adequate piece of dried hydrogel was accurately weighed, then immersed in 5 mL of PBS, and incubated in a water bath (DF-101S, Yuhua Instrument Co. LTD, Gongyi, China) at 37 °C with a shaking speed of 100 rounds per minute. The PBS was removed on days 7, 14, 21 and 28. After drying at 60 °C for 48 h, the samples were weighed again to determine the mass of the remaining hydrogel (*W_t_*). The degree of in vitro degradation of the hydrogel was calculated by the following weight ratio according to the relationship:

(3) $$\mathrm{Weight}\;\mathrm{ratio}\;(\%)=\frac{W_{t}}{W_{0}}\times 100$$

where $W_{0}$ and $W_{t}$ are the dry weight of the initial hydrogel and the remaining hydrogel after degradation at different time points, respectively.

### 2.7. Photothermal Conversion Properties of COL Hydrogel

Firstly, the absorbance of the hydrogel (wavelength range: 700–1100 nm) was studied by measuring the light absorbance of precursor solution (100 mg/mL, OD 38.5%, LAP 46%, CSP 15.5%) using a UV–Vis–NIR spectrophotometer (U-3900, Shimadzu, Kyoto, Japan). To simulate the photothermal effect of hydrogels in vivo, the following experiments were carried out. First, 100 μL of normal saline was pipetted into a 96-well cell culture plate, and 10 mg of the COL hydrogel was immersed in this solution to simulate the in vivo environment from an osmolarity point of view. Then, the hydrogels were irradiated with an 808 nm NIR laser at power densities of 0.5 W/cm^2^, 0.8 W/cm^2^, and 1.0 W/cm^2^ for 5 min. Normal saline was set as a control. The thermal imaging and temperature changes of the hydrogel during the abovementioned process were recorded every 10 s on average with an FLIRTM E60 (FLIR-E60, FLIR, OR, USA) infrared thermal imager. To investigate the photothermal stability of hydrogels, the COL hydrogel (10 mg) was immersed into a 96-well cell culture plate filled with 100 μL of normal saline. Using the same 808 nm NIR laser (0.8 W/cm^2^), well content was firstly irradiated for 5 min and naturally cooled for 5 min; this process was repeated for 6 cycles, followed by recording and comparing the temperature changes [29]. To explore the photothermal conversion efficiency (η) of the hydrogel according to Korgel’s method (Equation (4)), another cycle of laser irradiation was performed on the hydrogel or normal saline under the same conditions.

(4) $$\eta =\frac{hs\left(T_{max}-T_{surr}\right)-Q_{dis}}{I\left(1-10^{-A\left(\lambda \right)}\right)}\times 100$$

where h is the heat transfer coefficient of the system, s is the surface area of the irradiated hydrogel, $T_{max}$ is the maximum temperature of the hydrogel rising under irradiation, $T_{surr}$ is the ambient temperature during the photothermal experiment, I is the power of the 808 nm laser used, A(λ) is the absorbance value of the sample at 808 nm, and $Q_{dis}$ is the heat emitted to the environment during the experiment.

### 2.8. In Vitro Cytocompatibility of COL Hydrogels

The in vitro cytocompatibility of the COL hydrogels was analyzed using mouse fibroblasts (L929) as a model. Firstly, L929 cells were cultured in a constant-temperature cell incubator with a temperature of 37 °C and a CO_2_ content of 5% [30]. Subsequently, cells were divided into four groups and cultured with the COL hydrogel (hydrogel concentration: 0 (blank), 10 mg/mL, 20 mg/mL, and 50 mg/mL) for 24 h. Then, the hydrogel was removed, the cells were washed three times with PBS, and the culture was continued for 1 h with a CCK-8. The absorbance value at 450 nm was measured to calculate the cell viability. For the cell-staining observation using the inverted phase-contrast microscopy (Leica DM IL LED, Yuechanghang Technology Co., Ltd., Beijing, China), cells were washed three times with PBS and a Live/Dead double staining reagent was added to each well as per the standard procedures.

### 2.9. In Vitro Blood Compatibility of COL Hydrogels

The in vitro hemocompatibility of the COL hydrogels was evaluated via the hemolysis assay of mouse red blood cells (RBCs) as follows: 2 mL of blood was collected from anesthetized KM mice by cardiac puncturing, and mouse whole blood (2 mL) was centrifuged at 3000 rpm for 5 min to obtain RBCs [31,32]. Afterward, the obtained RBCs were washed three times with normal saline, and after each washing, they were centrifuged at 3000 rpm for 5 min. Finally, RBCs were stored in 100 mL of PBS. After mixing 1.2 mL of PBS with 0.3 mL of RBCs, a certain mass of lyophilized hydrogels was added to the above-mixed system (final hydrogel concentrations: 0 (negative control), 10, 20, and 50 mg/mL). The positive control group was composed of 1.2 mL of water and 0.3 mL of RBCs. The treated red blood cells were incubated in a 37 °C incubator for 2 h and then centrifuged (3000 rpm for 5 min) to obtain the supernatant. The absorbance of the supernatant at 541 nm was then measured. The hemolysis rate (HP) of erythrocytes after different treatments were calculated as follows:

(5) $$\mathrm{HP}\;\left(\%\right)=\frac{{\mathrm{OD}}_{\mathrm{sample}}-{\mathrm{OD}}_{\mathrm{negative}}}{{\mathrm{OD}}_{\mathrm{positive}}-{\mathrm{OD}}_{\mathrm{negative}}}\times 100$$

where OD_sample_ is the absorbance value of blood after hydrogel treatment, OD_negative_ is the absorbance value of blood after normal saline treatment, and OD_positive_ is the absorbance value of blood treated with DI water.

### 2.10. In Vivo Animal Tissue Safety of COL Hydrogels

For the in vivo animal tissue safety analysis of the COL hydrogels, nine KM mice (from Shanghai Experimental Animal Center, Shanghai, China) were randomly divided into three different groups after acclimatization: one control group and two experimental groups, with three animals per group (*n* = 3). The animal experiments were undertaken in strict accordance with the protocols authorized by the Experimental Animal Center of Changhai Hospital, Second Military Medical University, and the Ministry of Health. In brief, a 100 mg COL hydrogel was subcutaneously embedded in the experimental group of mice, and the healthy mice were not embedded in the control group. The experimental mice were euthanized on days 7 and 28 of feeding (the control mice were also euthanized on day 28). Then, blood was collected from the eyeballs of mice for serum biochemical indexes and routine blood analyses. At the same time, the skin tissues and major organs (heart, liver, spleen, lung, and kidney) of mice were removed from the embedding site and immersed in paraformaldehyde for H&E staining. The weight of the mice was recorded every 4 days during feeding.

### 2.11. Statistical Analysis

A one-way analysis of variance was conducted to assess the significance of experimental data by using the OriginPro 8.5 software package (OriginLab, Northampton, MA, USA). The probability (*p*) < 0.05, 0.01, and 0.001 are indicated by *, **, and ***, respectively.

## 3. Results and Discussion

### 3.1. Preparation and Characterization of COL Hydrogels

**Scheme 1:**
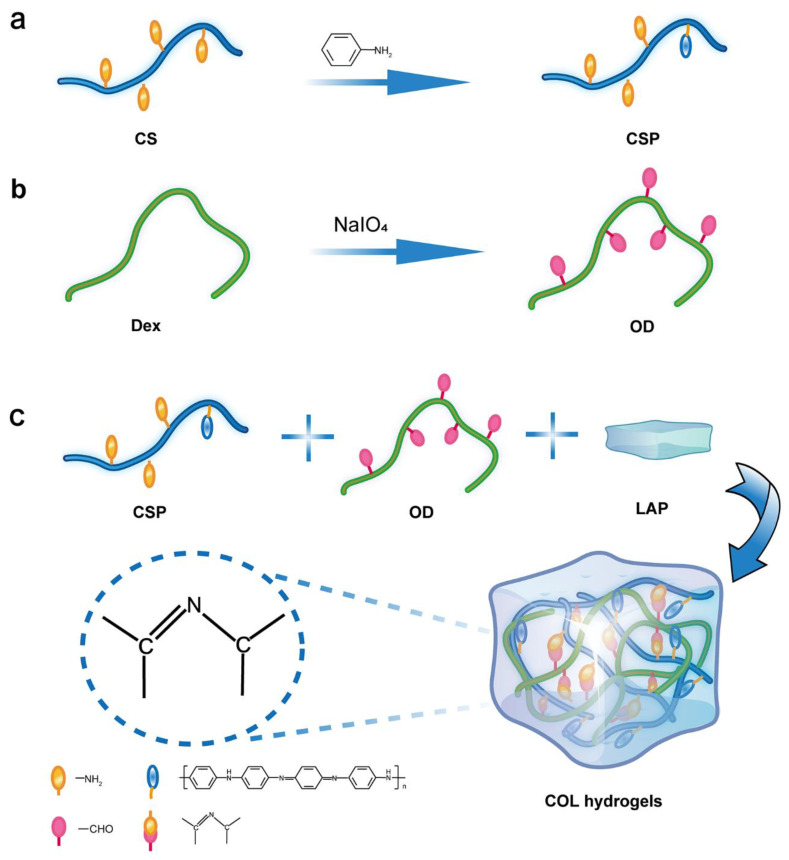
Schematic diagram of the hydrogel-forming process. (**a**) Preparation of polyaniline-grafted chitosan (CSP). (**b**) Preparation of oxidized dextran (OD). (**c**) Preparation of chitosan/polyaniline/laponite (COL) hydrogel.

**Figure 1:**
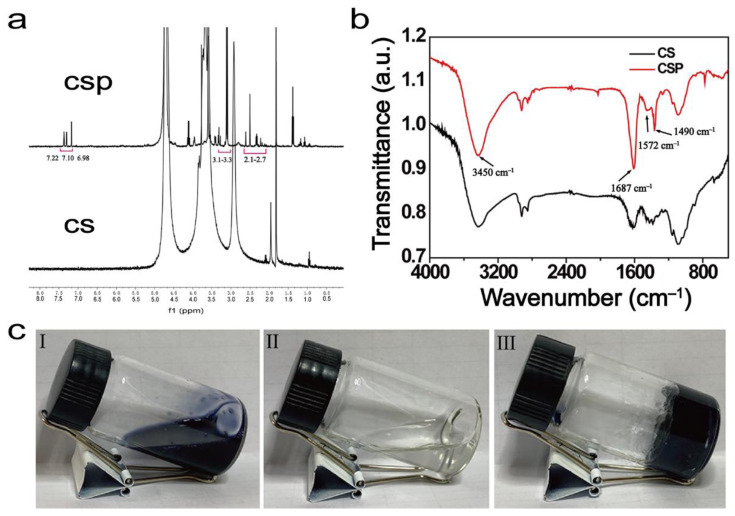
(**a**) ^1^H NMR spectra of gel polyaniline-grafted chitosan (CSP) and chitosan (CS) prepared in D_2_O at room temperature. (**b**) FTIR spectra for CSP and CS. (**c**) Digital photographs of the chitosan/polyaniline/laponite (COL) hydrogel formation process. I: CSP solution. II: Oxidized dextran/laponite (OD/LAP) solution. III: COL hydrogel.

The gelation process of OD and CSP is shown in Scheme 1. In this study, CS was firstly conjugated with aniline for a CSP. Then, Dex was oxidized to OD by NaIO_4_. Furthermore, LAP (an aluminosilicate clay) was physically doped in the hydrogel. CSP and OD can be cross-linked via Schiff base formation. The successful grafting of PAI onto CS was confirmed by ^1^H NMR (Figure 1a). In the spectra of CSP and CS, the peaks between 2.1 and 2.7 are the proton signals of C-H on the chitosan and the proton signals of the solvent for the detection (dimethyl sulfoxide). The peaks between 3.1 ppm and 3.3 ppm are the proton signals of hydroxyl groups on the chitosan [20]. In comparison to CS, CSP exhibited three new sharp peaks located at 6.98, 7.10 and 7.22 ppm in its ^1^H-NMR spectrum corresponding to the aromatic-type protons. This is direct evidence of the successfully grafting of PAI onto CS. In addition, the FTIR spectra of CS and CSP were comparatively studied (Figure 1b). In the CSP spectrum, the peak at 1687 cm^−1^ corresponds to -C=O in the partially deacetylated amide group -HNCOCH_3_ of chitosan, and the absorption peak of CSP at 3450 cm^−1^ is the stretching vibration peak of -O-H. Two absorption peaks at 1572 cm^−1^ and 1490 cm^−1^ appeared in the spectrum of CSP corresponding to the -C=C- stretching vibration on the quinone ring and the -C=C- stretching vibration on the benzene ring, respectively—both of which are characteristic peaks of polyaniline, indicating that aniline was successfully grafted on chitosan [33,34]. The FTIR results corroborated with the ^1^H NMR data indicating that PAI was successfully grafted onto the CS backbone. The degree of oxidation of OD determined by hydroxylamine hydrochloride titration was 76.28%. Digital photographs of the hydrogel formation process suggested that both OD/LAP and CSP were liquid substances with fluidity. Interestingly, the hydrogel formation could be carried out rapidly at room temperature. When the CSP solution was mixed with the OD solution, the mixture could complete the transition from sol to gel within 30 s to form a composite conductive hydrogel (Figure 1c).

**Figure 2:**
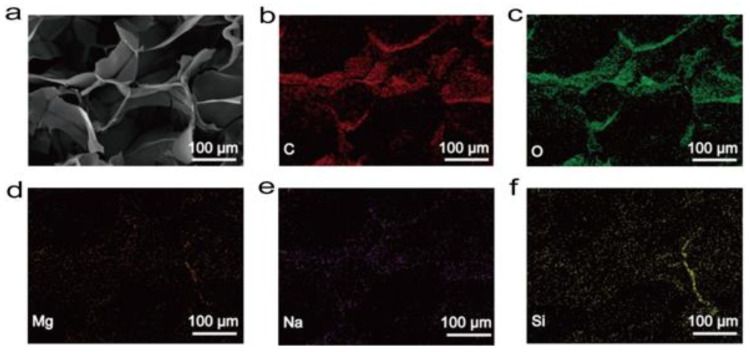
(**a**) SEM of chitosan/polyaniline/laponite (COL) hydrogels. Elemental distribution mappings of COL hydrogels ((**b**): C; (**c**): O; (**d**): Mg; (**e**): Na; (**f**): Si).

To examine the microstructural morphology and surface composition, the lyophilized hydrogels were investigated with SEM and elemental mappings, which experimentally showed an interconnected three-dimensional porous structure with a pore size of about 100–300 μm (Figure 2a). These pores were large enough to enable the migration of cells into the hydrogel and support the exchange of nutrients and metabolites. Meanwhile, the addition of CS, LAP, and PAI exerted no obvious effects on the pore size and surface roughness of the hydrogel. Moreover, elemental mapping results indicated a uniform distribution of Mg and Si, suggesting that LAP was uniformly doped in the gel (Figure 2b–f). It has been reported that LAP and other clay-containing materials have the potential to induce the osteogenic differentiation of bone marrow mesenchymal stem cells [35,36]. Therefore, LAP is expected to endow COL hydrogels with the significant ability to induce the osteogenic differentiation of stem cells.

### 3.2. Evaluation of the Swelling Properties of COL Hydrogels

**Figure 3:**
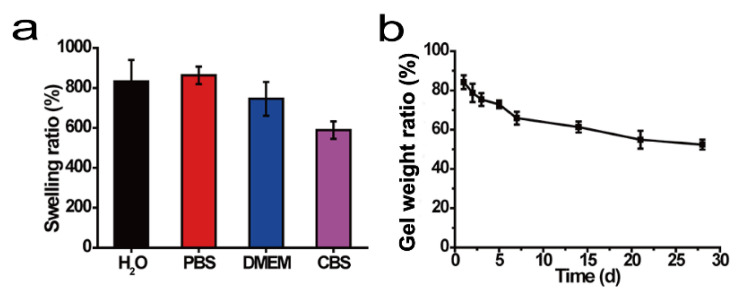
(**a**) Swelling properties of chitosan/polyaniline/laponite (COL) hydrogel in H_2_O, phosphate buffer solution (PBS), Dulbecco’s modified eagle medium (DMEM), and citrate buffer solution (CBS). (**b**) Degradation properties of COL hydrogel in PBS within 28 days.

The swelling of the COL hydrogel was measured at 37 °C, and the results are shown in Figure 3a. The COL hydrogel reached its equilibrium swelling within 24 h, with a swelling ratio of 832.9% in water, 863.4% in PBS, 745.5% in DMEM, and 588.8% in CBS, suggesting that the COL hydrogel has good swelling properties. The difference in swelling ratio in different solutions may be attributed to the different osmotic pressures of solutions. The osmotic pressures of water, CBS, PBS, and DMEM are different, so the swelling behaviors of hydrogels in these solutions are different. Water has the lowest osmotic pressure (practically equal to zero), so the hydrogel showed the highest swelling in water. This good swelling ability could beneficially contribute to the wide application prospects of the COL hydrogel in the biomedical field because the swollen hydrogel can mimic the structure of the natural extracellular matrix and provide support for cell proliferation.

### 3.3. Evaluation of the Degradation Properties of COL Hydrogels

The degradation study of the COL hydrogel in PBS was carried out at a temperature of 37 °C and a vibration speed of 100 rpm/min, and the results are shown in Figure 3b. The results experimentally demonstrated that the COL hydrogels degraded relatively rapidly in the first 7 days. Subsequently, the degradation rate of the COL hydrogel gradually decreased. The COL hydrogel lost about 30% of its mass within 7 days, and the mass–loss ratio was over 45% after 28 days. These findings indicated that the COL hydrogel is degradable, and this degradable property endows the hydrogel with a promising application future.

### 3.4. Photothermal Conversion Evaluation of COL Hydrogels

**Figure 4:**
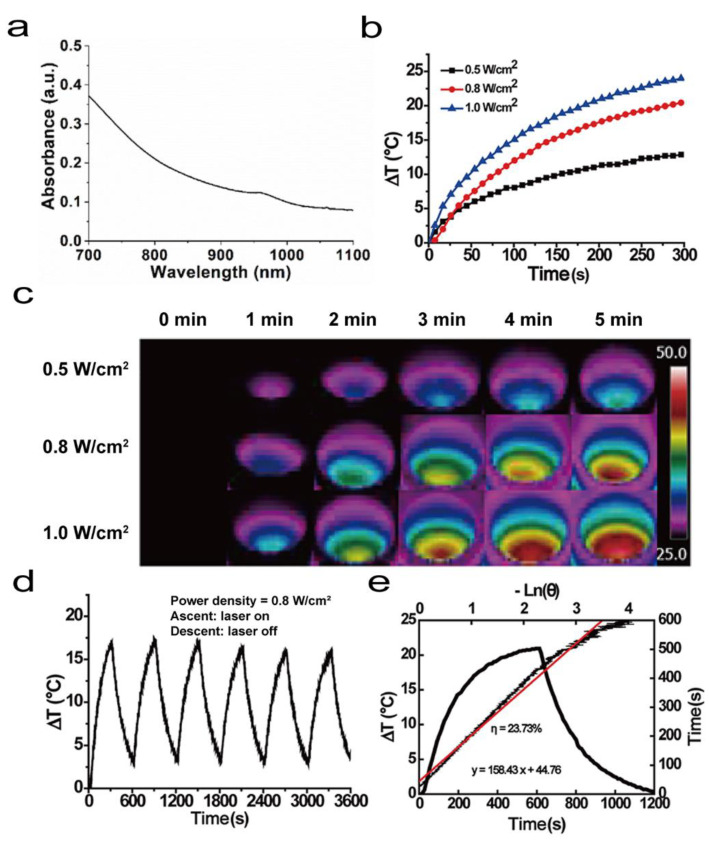
Photothermal conversion capacity of COL hydrogel. (**a**) NIR region of the UV–vis–NIR spectrum recorded for chitosan/polyaniline/laponite (COL) hydrogel. (**b**) Temperature rise curve of COL hydrogel containing saline at different laser densities of 808 nm for 5 min. (**c**) Thermogram of COL hydrogel corresponding to (**a**). (**d**) Photothermal stability of COL hydrogel over 6 lasers’ on/off cycles. (**e**) Temperature rise and fall curves and η of COL hydrogel.

Beyond the electrical conductivity, PAI further endowed the COL hydrogel with good photothermal conversion ability. Before exploring the photothermal conversion ability, the light absorption capacity of the COL hydrogel was measured with UV–vis–NIR spectroscopy. From Figure 4a, it can be seen that the COL hydrogel had a significant absorbance at a wavelength of 808 nm. The photothermal effect of the COL hydrogels was characterized by changing the laser density and illumination time (Figure 4b). Under the 5-min 808 nm NIR laser irradiation, the maximum temperature differences reached by the COL hydrogels were 12.9 °C (laser power density of 0.5 W/cm^2^), 20.6 °C (laser power density of 0.8 W/cm^2^), and 24.1 °C (laser power density of 1.0 W/cm^2^). These results are in good agreement with those arising from the corresponding infrared thermograms (Figure 4c), demonstrating the excellent photothermal properties of the COL hydrogels.

During the clinical trials, repeated treatments of the lesion site may be necessary since single-laser irradiation is not able to achieve the desired therapy efficiency. Therefore, a qualified platform should have good photothermal stability against repeated infrared light irradiation. To verify the photothermal stability of the COL hydrogel, continuous 808 nm NIR laser irradiation experiments were carried out. In the six lasers’ on/off cycles, the maximum temperature that the gel could reach had no decreasing trend and the ΔT fluctuated between 16 and 17.5 °C (Figure 4d); therefore, the COL hydrogel has good photothermal stability. Moreover, the photothermal conversion efficiency of the COL hydrogel was calculated to be 23.7% according to Korgel’s method (Figure 4e), which is higher than that of nanogels made of poly(acrylic acid-b-N-isopropylamide-b-acrylic acid/polypyrrole (20.2%) [37], PBIPDI-TT (a new thieno-isoindigo derivative dye and its D−A polymer; 20.3%) [38], and polyethylene glycol-coated copper nanowires (12.5%) [39]. The photothermal conversion data experimentally proved that the COL hydrogel could be used as a PTT platform.

### 3.5. Evaluation of In Vitro Cytocompatibility of COL Hydrogels

**Figure 5:**
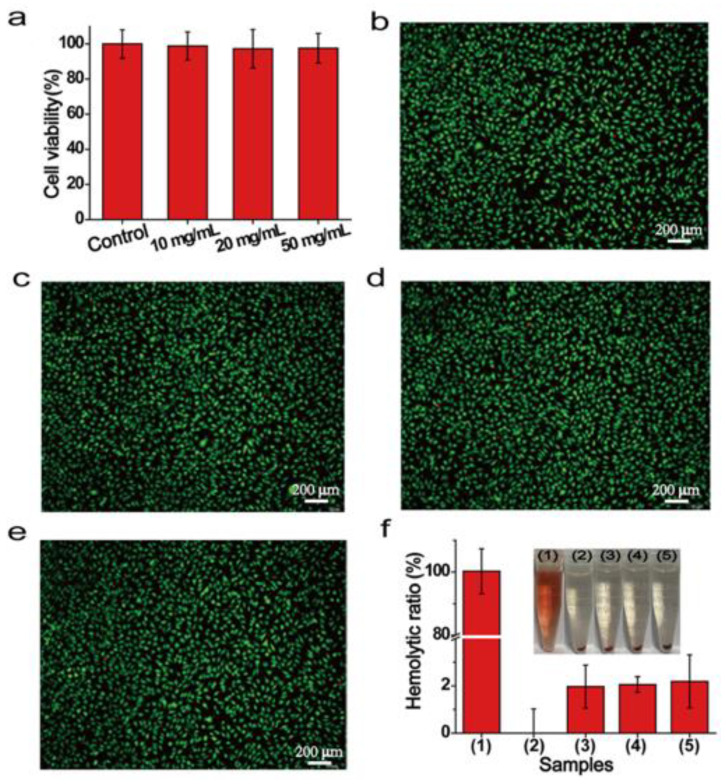
The safety of chitosan/polyaniline/laponite (COL) hydrogel in vitro. (**a**) Mouse fibroblasts (L929) cell viabilities after being cultured with COL hydrogel. Photographs of Live/Dead staining of cells in (**b**) control group, (**c**) 10 mg/mL hydrogel, (**d**) 20 mg/mL hydrogel, and (**e**) 50 mg/mL hydrogel. (**f**) Hemolysis ratios of mouse red blood cells (RBCs) cultured with COL hydrogel; the inset is the corresponding centrifuged mRBC photograph ((1): deionized (DI) water; (2): phosphate buffer solution (PBS); (3): 10 mg/mL hydrogel; (4): 20 mg/mL hydrogel; and (5): 50 mg/mL hydrogel).

By selecting the L929 cell as a model, the cytocompatibility of the COL hydrogel was evaluated in vitro. The cell viability of the control group was appointed as 100% to exclude the influence of environmental factors. After L929 cells were co-cultured with 10 mg/mL, 20 mg/mL, and 50 mg/mL of hydrogels for 24 h, the cell viability was as high as 98.8%, 97.2%, and 97.4%, respectively. The CCK-8 results showed that the COL hydrogel had good cytocompatibility (Figure 5a). After the CCK-8 assay, we observed images of cells stained with the Live/Dead kit using an inverted phase-contrast microscope. It can be seen that the green fluorescence representing live cells occupied most of the area in the control and experimental groups, while the red fluorescence representing dead cells was negligible (Figure 5b–e). Moreover, the cell morphology of the experimental group was not significantly different than that of the control group. This aspect is consistent with the experimental results of CCK-8, both types of results demonstrating the good cytocompatibility of the COL hydrogel.

### 3.6. Evaluation of In Vitro Hemocompatibility of COL Hydrogels

By employing RBCs as another model, the in vitro biocompatibility of the COL hydrogel was further assessed. In this assay, RBCs were incubated with different concentrations of hydrogels for 2 h and centrifuged. The harvested supernatant was collected and its absorbance of 541 nm was tested to calculate the HP. As shown in Figure 5f, using distilled-water-treated blood cells as a positive control, the HP was calculated as about 100%. Taking the blood cells treated with normal saline as the negative control, the HP was calculated to be zero, and the HPs of blood cells treated with 10 mg/mL, 20 mg/mL, and 50 mg/mL of the COL hydrogel were well-below the threshold for hemolysis, that is, 5%, suggesting that the COL hydrogel showed good hemocompatibility. The corresponding images of blood cells after centrifugation are shown in the inset of Figure 5f. In the images, the red color represents the occurrence of hemolysis. The diagram in Figure 5f quantitatively indicates that the COL hydrogel has good blood compatibility.

### 3.7. Evaluation of In Vivo Biocompatibility of COL Hydrogels

**Figure 6:**
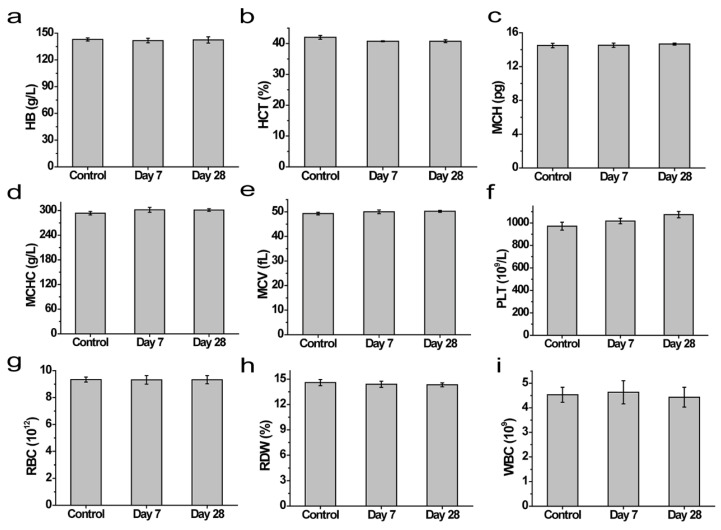
(**a**–**i**) Routine blood results for different days in mice subcutaneously embedded in hydrogel or blank (control).

**Figure 7:**
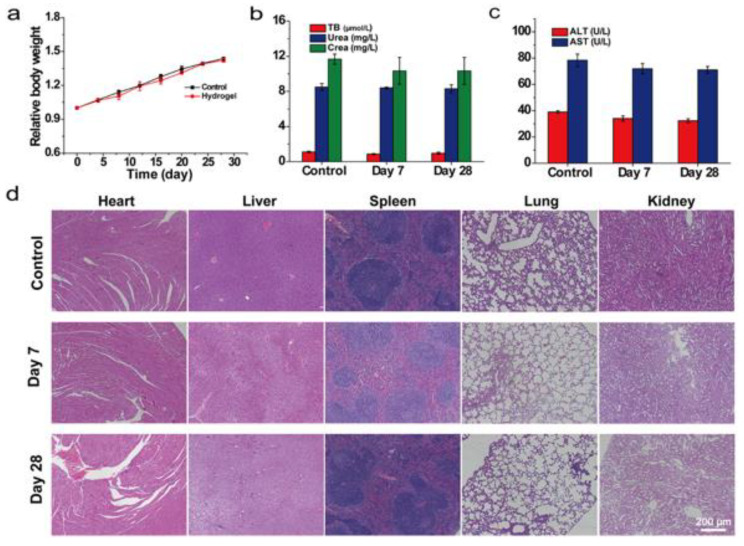
(**a**) Relative bodyweight of Kunming (KM) mice from different groups. (**b**,**c**) Blood biochemistry parameters of KM mice treated with or without chitosan/polyaniline/laponite (COL) hydrogels. (**d**) H&E staining of the major organs of KM mice treated with or without COL hydrogels.

In addition to cytocompatibility, an excellent biomedical hydrogel material needs to have good biocompatibility at the animal level. Therefore, we further verified the biosafety of the COL hydrogels on KM mice. The routine blood parameters (including white blood cell count (WBC), red cell distribution width (RDW), red blood cell count (RBC), hemoglobin (HB), hematocrit (HCT), mean erythrocyte volume (MCV), platelet count (PLT), mean RBC hemoglobin content (MCH), and mean RBC hemoglobin concentration (MCHC)) of the mice on the 7th and 28th days were similar to the data of the control group, and no obvious physiological abnormalities could be identified (Figure 6a–i). Moreover, compared to healthy mice in the control group, the bodyweight of the COL hydrogel-embedded mice did not significantly fluctuate during the feeding period (Figure 7a). The results of the serum biochemical study showed no significant differences between the control and experimental groups (Figure 7b,c). Finally, pathological sections from skin tissue and main organs were taken from the embedding site for H&E staining, which showed that the COL hydrogel exerted no obvious damage to the skin tissue and main organs after 7 or 28 days of subcutaneous embedding. The in vivo biocompatibility evaluation showed that the COL hydrogel had no obvious acute and chronic side effects on major organs, thus ensuring the safety of the COL hydrogel in vivo (Figure 7d).

## 4. Conclusions

In summary, we successfully designed a multifunctional COL hydrogel with good biocompatibility and photothermal conversion capacity using CS, PAI, LAP, and OD as raw materials. The formation of the hydrogel via the Schiff base reaction between CSP and OD was carried out at room temperature. It was observed with SEM that the hydrogel had a regular three-dimensional porous structure. We then studied the swelling and degradation behaviors, as well as photothermal conversion. The research results showed that the composite hydrogel was degradable and had good photothermal conversion performance. Furthermore, the CCK-8 experiment confirmed that the COL hydrogel was biocompatible and the cell viability was as high as 98.8%, 97.2%, and 97.4% after being cultured with 10 mg/mL, 20 mg/mL, and 50 mg/mL hydrogels, respectively, for 24 h. The in vivo biocompatibility was confirmed at the mice level. In conclusion, this multifunctional composite hydrogel will provide a new reference for the tumor therapy and tissue regeneration application of hydrogels.

## Data Availability

The data that support the findings of this study are available from the corresponding author upon reasonable request.
